# Headache following head injury: a population-based longitudinal cohort study (HUNT)

**DOI:** 10.1186/s10194-018-0838-2

**Published:** 2018-01-22

**Authors:** Lena Hoem Nordhaug, Knut Hagen, Anne Vik, Lars Jacob Stovner, Turid Follestad, Torunn Pedersen, Gøril Bruvik Gravdahl, Mattias Linde

**Affiliations:** 1 Department of Neuromedicine and Movement Science (INB), Faculty of Medicine and Health Sciences, Norwegian University of Science and Technology, Postbox 8905, 7491 Trondheim, Norway; 2 Department of Neurosurgery, St. Olavs University Hospital, Trondheim, Norway; 3 Norwegian Advisory Unit on Headaches, St. Olavs University Hospital, Trondheim, Norway; 4 Department of Public Health and Nursing, Faculty of Medicine and Health Sciences, Norwegian University of Science and Technology, Trondheim, Norway; 5 Division of Mental Health and Addiction, Oslo University Hospital, Oslo, Norway

**Keywords:** Headache attributed to head injury, Head injury, Traumatic brain injury, Secondary headache disorders, Post-traumatic headache, Population-based

## Abstract

**Background:**

Headache is the most frequent symptom following head injury, but long-term follow-up of headache after head injury entails methodological challenges. In a population-based cohort study, we explored whether subjects hospitalized due to a head injury more often developed a new headache or experienced exacerbation of previously reported headache compared to the surrounding population.

**Methods:**

This population-based historical cohort study included headache data from two large epidemiological surveys performed with an 11-year interval. This was linked with data from hospital records on exposure to head injury occurring between the health surveys. Participants in the surveys who had not been hospitalized because of a head injury comprised the control group. The head injuries were classified according to the Head Injury Severity Scale (HISS). Multinomial logistic regression was performed to investigate the association between head injury and new headache or exacerbation of pre-existing headache in a population with known pre-injury headache status, controlling for potential confounders.

**Results:**

The exposed group consisted of 294 individuals and the control group of 25,662 individuals. In multivariate analyses, adjusting for age, sex, anxiety, depression, education level, smoking and alcohol use, mild head injury increased the risk of new onset headache suffering (OR 1.74, 95% CI 1.05–2.87), stable headache suffering (OR 1.70, 95% CI 1.15–2.50) and exacerbation of previously reported headache (OR 1.93, 95% CI 1.24–3.02). The reference category was participants without headache in both surveys.

**Conclusion:**

Individuals hospitalized due to a head injury were more likely to have new onset and worsening of pre-existing headache and persistent headache, compared to the surrounding general population. The results support the entity of the ICHD-3 beta diagnosis “persistent headache attributed to traumatic injury to the head”.

## Background

Headache often has a major impact on the lives of the individuals affected, and constitutes a large social and economic burden for the global society [1–3].

Likewise, head injury is an important global health issue and a major cause of morbidity [4, 5]. Headache is the most frequent symptom following head injury, and it is manifested both as new onset and worsening of pre-existing headache [6–9]. The international classification of headache disorders (third edition, ICHD-3 beta) defines headache attributed to head injury (HAIH) as a headache with no defining clinical characteristics that starts within seven days of injury [10]. Persistent HAIH is of greater than 3 months’ duration [10]. Long-term follow-up of headache after head injury entails methodological challenges. To investigate a causal relationship between head injury and subsequent headache, a control group for comparison is vital, preferably through a population-based design. There are, to our knowledge, only two other population-based, controlled studies on this subject and their findings are inconsistent [11, 12]. Moreover, both studies have methodological limitations [11, 12]. Headache prevalence and severity have been reported to be greater in those with mild head injury compared to those with more severe head injury [13, 14]. This inverse dose-response relationship is paradoxical and needs further investigation.

In a previous population-based historical cohort study, which was based on the third wave of the Nord-Trøndelag Health Study (HUNT), we evaluated the relationship between previous head injury and headache phenotype [15]. The primary aim of the current study was to analyze headache data for those who participated in both the second and third waves of the HUNT Study, evaluating the impact on new onset headache or exacerbation of headache due to head injuries in a population with known pre-injury headache status, taking into account the head injury severity.

## Methods

### Study design

This historical cohort study included data on headache from the second and third HUNT surveys, two large epidemiological surveys performed with an 11-year interval. Headache data was linked with data from hospital records on exposure to head injury occuring between the surveys. The exposed group consisted of study participants hospitalized due to a head injury, the remaining participants were used as controls.

### The HUNT-surveys

The HUNT Study is a longitudinal cohort study in which all inhabitants ≥20 years of age in Nord-Trøndelag were invited to participate. Participants were examined three times. The two last surveys, HUNT2 (1995–1997) and HUNT3 (2006–2008) covered a large number of health-related items. Details of these comprehensive surveys, including non-respondents, are described elsewhere [16, 17].

### Headache categories

Both HUNT2 and HUNT3 questionnaires included the screening question “Have you suffered from headache during the last 12 months?” The answers to the screening questions were used to categorize the responders into four mutually exclusive groups with regard to headache suffering at the two time points: Stable non-sufferers (headache-free in both studies), past sufferer (headache in HUNT2 but not in HUNT3), stable headache sufferer (headache in both studies) and new sufferer (headache in HUNT3, but not in HUNT2).

Participants answering “yes” to the headache screening question also reported their headache frequency. This enabled categorization of the responders with headache suffering in both surveys into three mutually exclusive groups: less frequent headache (in HUNT3 compared to HUNT2), stable headache frequency (same headache frequency in HUNT3 as in HUNT2), more frequent headache (in HUNT3 compared to HUNT2). Alternatives available for the headache frequency question were < 7, 7–14 and > 14 days/month.

To examine exacerbation or improvement in headache status between HUNT2 and HUNT3 we merged the frequency variable with the screening question so that each participant could be categorized in one of the following four groups: no headache suffering, headache suffering < 7 days/month, headache suffering 7–14 days/month, headache suffering > 14 days/month. Exacerbation of headache was defined as new onset of headache or increased frequency of previously reported headache. Improvement was defined as absence of or decrease in frequency of previously reported headache. Stable headache frequency was defined as headache suffering in both surveys with the same headache frequency in both.

Pre-existing headache was classified into two mutually exclusive groups: Migraine and non-migrainous headache. The approach used in determining headache subtype is described and validated elsewhere [18].

The validities of the headache questionnaires in HUNT2 and HUNT3 have been reported previously [18, 19]. For any headache suffering in HUNT2, the sensitivity was 85% and specificity 83% (kappa 0.57, 95% CI 0.41–0.73). For any headache suffering in HUNT3, the sensitivity was 88% and specificity 86% (kappa 0.70, 95% CI 0.61–0.79). A personal interview by a neurologist was used as gold standard.

### Head injury data collection

All participants who had answered the headache screening question in HUNT3 and who had also been hospitalized in the region due to a head injury during the period 1988–2008 were identified in 2012 by a computer-based search. Details on how this was performed have been reported previously [15].

Information regarding the head injuries was collected from medical records. If the same individual had more than one head injury within the period, up to three of the most recent head injuries were recorded. The head injuries were classified according to the Head Injury Severity Scale (HISS) [20].

Only participants who answered the headache screening question in both HUNT2 and HUNT3 were of interest for analyses. Participants with head injuries between participation in HUNT2 and HUNT3 were included in the exposed group. Participants with head injuries before HUNT2 were excluded.

### Other measurements

The HUNT2 and HUNT3 surveys included many health-related items, and in the present study we used the following information about the participants in addition to headache status: age, sex, duration of education, smoking habits, total Hospital Anxiety and Depression Scale (HADS) score, self-reported health, BMI and CAGE score. The HADS is a fourteen item scale used to determine levels of anxiety and depression. The CAGE questionnaire is a widely used screening instrument for potential alcohol problems [21].

### Statistical analysis

Demographic data for individuals with and without head injury are presented as means with standard deviations (continuous variables) and percentages (categorical data). In multivariate analyses, using multinomial logistic regression, we first examined the association between head injury and relative headache status in HUNT3 versus HUNT2 with regard only to suffering from headache and then the association between head injury and relative headache status in HUNT3 versus HUNT2 with regard both to suffering from headache and change in headache frequency. The results are presented as odds ratios (OR) with 95% confidence intervals (CI). OR’s for which the 95% CI did not include 1 were considered statistically significant. Stable non-sufferers were used as reference category. The associations were investigated both with head injury as a binary variable (yes/no) and with head injury in four categories according to head injury severity (no head injury/minimal/mild/moderate head injury).

In the multivariate analyses we initially adjusted for age and sex. Subsequently, we also added the following potential confounding factors retrieved from the HUNT2 dataset: duration of education (≤ 9, 10–12 and ≥13 years) as proxy for socioeconomic status, daily smoking (yes/no), total HADS score (categorized from a continuous variable into three categories with score ≤ 16, 17–21 and ≥22) and CAGE score (0 or ≥1). In the logistic regression analyses missing data were handled by listwise deletion. Linearity of the continuous variables with respect to the logit of the dependent variable was assessed via the Box-Tidwell procedure [22]. The continuous variable age was found to fail the assumption of linearity and was split into categories with 10 year intervals. We investigated potential interactions between all covariates and head injury by including the product of the two variables into the multinomial logistic regression analyses. The interaction coefficients were tested using Wald statistics, with *p*-values less than 0.05 considered statistically significant. Because headache disorders in general are highly dependent on sex [17], separate analyses after stratifying for sex are common in research concerning headache. Consequently we chose to also do separate analyses after stratifying for sex. No formal adjustment for multiple testing was made.

The study was approved by the Regional Committee for Medical and Health Research Ethics and by the HUNT Research Centre.

## Results

### Participants

**Fig. 1:**
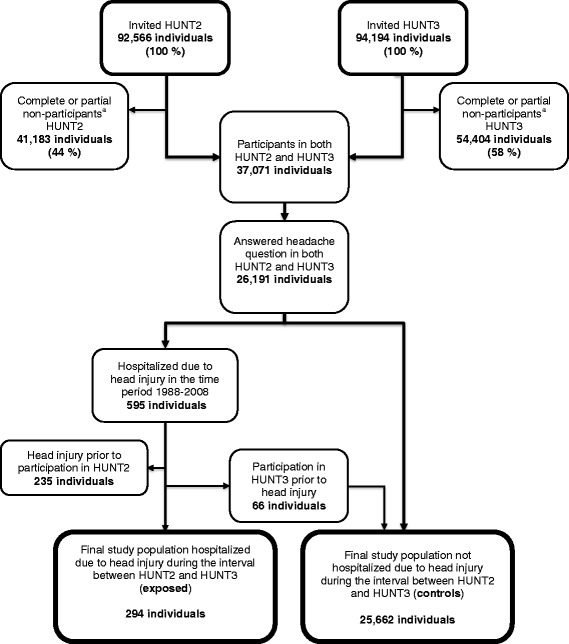
Flow of participants through the HUNT2 and HUNT3 study and selection of head injury patients. ^a^Complete non-participants: Answering no questionnaire. Partial non-participants: Answering part of the questinonnaire, but not the headache screening question. Abbreviations: HUNT = the Nord-Trøndelag Health Study

The flow of participants through the different stages of the study is presented in Fig. 1. A total of 294 participants had been hospitalized due to a head injury during the 11 year time period between the two surveys (exposed). The remaining 25,662 individuals were not hospitalized for head injury during this period (unexposed). Demographic data for the two groups are presented in Table 1. Prevalence of headache in HUNT2 was similar in the two groups (43.9% versus 41.7%, χ^2^ (1) = 0.575, *p* = 0.45).

**Table 1:** Demographics and health related variables

	Head injury population	Controls
Variable		
Total number of subjects	294	25,662
Age at participation in HUNT2 (years) (mean ± SD)	48.4 ± 14.7	47.3 ± 13.1
Age at injury (first injury) (years) (mean ± SD)	53.8 ± 15.2	
Age at participation in HUNT3 (years) (mean ± SD)	59.6 ± 14.7	58.5 ± 13.1
Time from head injury (first injury) to HUNT3 (years) (mean ± SD)	4.9 ± 3.2	
Female	137 (46.5)	14,639 (57.0)
Duration of education (HUNT2) (n (%))		
≤ 9 years	82 (27.9)	7535 (29.4)
10–12 years	135 (45.9)	11,663 (45.4)
≥ 13 years	73 (24.8)	6007 (23.4)
HADS score (mean ± SD) (HUNT2)	8.8 ± 5.7	7.4 ± 5.4
HADS score (mean ± SD) (HUNT3)	8.5 ± 6.0	7.3 ± 5.4
BMI (mean ± SD) (HUNT2)	26.2 ± 3.9	26.0 ± 3.6
BMI (mean ± SD) (HUNT3)	27.4 ± 4.3	27.4 ± 4.3
Daily smoking (HUNT2) (n (%))	62 (21.1)	6278 (24.5)
Daily and occasionally smoking (HUNT3) (n (%))	73 (24.8)	5338 (20.8)
Self-reported health poor or less than good (HUNT2) (n (%))	82 (27.9)	5451 (21.2)
Self-reported health poor or less than good (HUNT3) (n (%))	102 (34.7)	6883 (26.8)
Headache sufferer (n (%))		
HUNT2	129 (43.9)	10,697 (41.7)
HUNT3	111 (37.8)	8492 (33.1)
CAGE^a^ ≥ 1 (HUNT2) (n (%))	56 (19.0)	3721 (14.5)
CAGE^a^ ≥ 1 (HUNT3) (n (%))	58 (19.7)	3834 (14.9)

Abbreviations: *HADS* Hospital Anxiety and Depression Scale, *BMI* Body Mass Index

^a^*CAGE* Modified Norwegian version of the CAGE alcohol-screening instrument

### Head injuries

Among the 294 individuals with head injuries, 11 experienced two head injuries, adding up to a total of 305 incidents. Taking into account only the first head injury (of those with more than one), 11.9% of the head injuries were minimal, 71.8% were mild, 10.9% were moderate and 5.4% were unclassifiable. There were no severe head injuries. The most common injury mechanism was falling (55.1%), followed by traffic accidents (28.6%) and assault (1.7%).

A CT scan was performed in 56.4% (*n* = 172), an MRI in 1.0% (*n* = 3) and a plain X-ray of the head in 3.6% (*n* = 11) of the cases. The scans revealed traumatic pathology in 16.0% (*n* = 47). In total, 9.2% (*n* = 27) of all patients had intracranial pathology. 9.9% (*n* = 29) had cranial fractures (revealed either by imaging or clinical findings), and 5.1% (*n* = 15) had both cranial fracture and intracranial pathology.

### Headache categories

Among the 25,956 partcipants, 12,830 (49.4%) were headache free in HUNT2 and HUNT3 (stable non-suffering), 6303 (24.3%) suffered from headache in both surveys (stable headache suffering), 4523 (17.4%) reported headache in HUNT2 only (past headache suffering) and 2300 participants (8.9%) had no headache in HUNT2, but reported to suffer from headache in HUNT3 (new headache suffering).

Among the 129 participants with head injury and pre-existing headache, 46 (35.7%) suffered from migraine, 57 (44.2%) from non-migrainous headache and 26 (20.2%) were unclassifiable.

Individuals with mild head injury were more likely to have new onset of headache (OR 1.74, 95% CI 1.05–2.87) and stable headache suffering (OR 1.70, 95 CI 1.15–2.50) compared to controls (Table 2).

**Table 2:** Multivariate regression analyses of the associations between head injury and relative headache status in HUNT3 versus HUNT2 with regard to suffering from headache only

		All		Men	Women
		Adjustment for age and sex	Complete adjustment^a^	Complete adjustment^a^	Complete adjustment^a^
	N	N (OR, 95% CI)	N (OR, 95% CI)	N (OR, 95% CI)	N (OR, 95% CI)
Past headache suffering					
No head injury (reference)	25,662	4474 (ref.)	4474 (ref.)	1603 (ref.)	2871 (ref.)
Any head injury	294	49 (1.21, 0.87–1.69)	49 (1.23, 0.85–1.78)	24 (1.35, 0.82–2.21)	25 (1.06, 0.60–1.86)
Minimal head injury	35	7 (1.15, 0.47–2.80)	7 (0.94, 0.33–2.70)	1 (0.50, 0.06–4.14)	6 (1.17, 0.33–4.18)
Mild head injury	211	36 (1.35, 0.91–2.00)	36 (1.39, 0.89–2.15)	18 (1.53, 0.85–2.75)	18 (1.19, 0.61–2.32)
Moderate head injury	32	5 (1.13, 0.41–3.13)	5 (1.24, 0.43–3.54)	4 (1.41, 0.44–4.49)	1 (0.93, 0.08–10.52)
Stable headache suffering					
No head injury (reference)	25,662	6223 (ref.)	6223 (ref.)	1833 (ref.)	4390 (ref.)
Any head injury	294	80 (1.51, 1.13–2.03)	80 (1.55, 1.12–2.14)	37 (1.65, 1.06–2.56)	43 (1.37, 0.85–2.20)
Minimal head injury	35	10 (1.20, 0.53–2.70)	10 (0.96, 0.38–2.42)	4 (1.22, 0.32–4.60)	6 (0.78, 0.21–2.86)
Mild head injury	211	56 (1.60, 1.12–2.27)	56 (1.70, 1.15–2.50)	27 (1.91, 1.13–3.22)	29 (1.42, 0.80–2.51)
Moderate head injury	32	9 (1.58, 0.66–3.75)	9 (1.45, 0.57–3.70)	4 (0.90, 0.24–3.34)	5 (2.54, 0.45–14.30)
New headache suffering					
No head injury (reference)	25,662	2269 (ref.)	2269 (ref.)	974 (ref.)	1295 (ref.)
Any head injury	294	31 (1.44, 0.96–2.15)	31 (1.37, 0.88–2.15)	21 (1.89, 1.12–3.21)	10 (0.70, 0.29–1.69)
Minimal head injury	35	1 (0.33, 0.04–2.47)	1 (0.38, 0.05–2.90)	1 (0.81, 0.10–6.75)	0 (−)
Mild head injury	211	27 (1.86, 1.20–2.90)	27 (1.74, 1.05–2.87)	17 (2.31, 1.26–4.23)	10 (1.02, 0.41–2.52)
Moderate head injury	32	3 (1.20, 0.34–4.22)	3 (1.30, 0.36–4.65)	3 (1.75 (0.48–6.42)	0 (−)

One analysis was done with head injury as a binary variable (no head injury/any head injury) and a separate analysis was done with head injury in four categories according to head injury severity (no head injury/minimal head injury/mild head injury/moderate head injury). The head injury severity was classified according to the Head Injury Severity Scale (HISS)

Reference category: Stable non-sufferer (absence of headache suffering in both surveys)

Abbreviations: *OR* Odds ratio, *CI* Confidence interval

^a^Analyses are adjusted for age, sex, duration of education, daily smoking, CAGE score ≥ 1 and HADS-score

Table 3 demonstrates change in monthly headache frequency between HUNT2 and HUNT3 among participants suffering from headache in both surveys. In the head injury population 15.0% had more frequent headache in HUNT3 than in HUNT 2, in the control population the corresponding proportion was 12.2%.

**Table 3:** Change in monthly headache frequency between HUNT2 and HUNT3 among participants suffering from headache in both surveys

		N (%), head injury population	N (%), controls
HUNT2	HUNT3		
Decreased headache frequency^a^		10 (12.5)	939 (15.1)
> 14 days	1–14 days	2 (2.5)	269 (4.3)
7–14 days	< 7 days	8 (10.0)	670 (10.8)
Stable headache frequency^b^		55 (68.8)	4234 (68.0)
< 7 days	< 7 days	46 (57.5)	3833 (61.6)
7–14 days	7–14 days	4 (5.0)	257 (4.1)
> 14 days	> 14 days	5 (6.3)	144 (2.3)
Increased headache frequency^c^		12 (15.0)	757 (12.2)
< 7 days	7–30 days	10 (12.5)	633 (10.2)
7–14 days	> 14 days	2 (2.5)	124 (2.0)
Missing		3 (3.8)	293 (4.7)
Total		80 (100.0)	6223 (100.0)

^a^Less frequent headache (days/month) in HUNT3 compared to HUNT2

^b^Same headache frequency (days/month) in HUNT3 as in HUNT2

^c^More frequent headache (days/month) in HUNT3 compared to HUNT2

There was a significant association between exacerbation of headache and head injury (Table 4). There was a nearly doubled odds of exacerbation of headache among those exposed to mild head injury than among the controls (OR 1.93, 95% CI 1.24–3.02) (Table 4). Individuals with head injury were also more likely to have stable headache suffering with unchanged frequency from HUNT2 to HUNT3 than the controls (Table 4). There was no significant relationship between moderate head injury and any of the headache trajectories (Tables 2 and 4).

**Table 4:** Multivariate regression analyses of the associations between head injury and relative headache status in HUNT3 versus HUNT2 with regard both to suffering from headache as well as to change in headache frequency

		All		Men	Women
		Adjustment for age and sex	Complete adjustment^a^	Complete adjustment^a^	Complete adjustment^a^
	N	N (OR, 95% CI)	N (OR, 95% CI)	N (OR, 95% CI)	N (OR, 95% CI)
Improvement of headache status^1^					
No head injury (reference)	25,662	5413 (ref.)	5413 (ref.)	1852 (ref.)	3561 (ref.)
Any head injury	294	59 (1.22, 0.89–1.68)	59 (1.21, 0.85–1,72)	28 (1.30, 0.81–2.08)	31 (1.06, 0.63–1.80)
Minimal head injury	35	12 (1.62, 0.76–3.44)	12 (1.35, 0.56–3.24)	2 (0.76, 0.15–3.82)	10 (1.65, 0.54–4.98)
Mild head injury	211	40 (1.26, 0.86–1.84)	40 (1.27, 0.83–1.95)	20 (1.39, 0.79–2.47)	20 (1.09, 0.58–2.07)
Moderate head injury	32	6 (1.16, 0.44–3.02	6 (1.23, 0.45–3.31)	5 (1.51, 0.51–4.42)	1 (0.66, 0.06–7.63)
Stable headache suffering and frequency^2^					
No head injury (reference)	25,662	4234 (ref.)	4234 (ref.)	1302 (ref.)	2932 (ref.)
Any head injury	294	55 (1.55, 1.11–2.15)	55 (1.60, 1.12–2.28)	28 (1.86, 1.16–2.99)	27 (1.30, 0.76–2.21)
Minimal head injury	35	5 (0.88, 0.32–2.45)	5 (0.71, 0.23–2.25)	3 (1.37, 0.33–5.68)	2 (0.28, 0.03–2.39)
Mild head injury	211	40 (1.71, 1.15–2.53)	40 (1.83, 1.20–2.79)	20 (2.14, 1.22–3.76)	20 (1.48, 0.79–2.77)
Moderate head injury	32	5 (1.26, 0.44–3.61)	5 (1.06, 0.33–3.40)	3 (0.87, 0.19–4.02)	2 (1.40, 0.18–10.82)
Exacerbation of headache status^3^					
No head injury (reference)	25,662	3026 (ref.)	3026 (ref.)	1181 (ref.)	1845 (ref.)
Any head injury	294	43 (1.55, 1.09–2.21)	43 (1.52, 1.02–2.25)	26 (1.87, 1.14–3.05)	17 (1.08, 0.56–2.08)
Minimal head injury	35	1 (0.24, 0.03–1.83)	1 (0.26, 0.03–2.03)	1 (0.62, 0.07–5.16)	0 (−)
Mild head injury	211	37 (2.00, 1.35–2.98)	37 (1.93, 1.24–3.02)	22 (2.40, 1.37–4.21)	15 (1.37, 0.66–2.85)
Moderate head injury	32	5 (1.56, 0.55–4.41)	5 (1.65, 0.57–4.79)	3 (1.42, 0.39–5.22)	2 (2.09, 0.27–16.16)

One analysis was done with head injury as a binary variable (no head injury/any head injury) and a separate analysis was done with head injury in four categories according to head injury severity (no head injury/minimal head injury/mild head injury/moderate head injury). The head injury severity was classified according to the Head Injury Severity Scale (HISS)

Reference category: Stable non-sufferer (absence of headache suffering in both surveys)

Abbreviations: *OR* Odds ratio, *CI* Confidence interval

^1^Improvement of headache status: Absence of previously reported headache or decrease in its frequency

^2^Stable headache suffering and frequency: Headache suffering in both HUNT2 and HUNT3 with the same frequency in both studies

^3^Exacerbation of headache status: New onset of headache or increased frequency of previously reported headache

^a^Analyses are adjusted for age, sex, duration of education, daily smoking, CAGE score ≥ 1 and HADS-score

There were no significant associations between head injury and past headache suffering or improvement of headache status.

No consistent significant interaction was observed between head injury and any of the covariates. The only significant interaction observed was for smoking and only in the case of improvement of headache.

Although no significant interaction was observed between sex and head injury, we did separate analyses for males and females for all headache trajectories. In these separate analyses, significant associations were found only for men (Tables 2 and 4).

## Discussion

This is the first study which presents population based data on headache occurrence after head injury with known pre-injury headache status. Our main finding was that exposure to head injury increased the risk of new onset headache suffering and exacerbation of headache. Also, head injury was positively associated with stable headache suffering, which means that headache was less likely to improve. This confirms findings from several studies with less reliable study designs during the last years [9, 23, 24], but contrast with an earlier population-based study, which did not find an association between previous head injury and headache [12]. However, in that study there was a 22-year interval between the exposure to trauma and inquiry about headache [12].

The data were analyzed with regard to sex differences, since both prevalences of primary headache, as well as injuries to the head, are known to differ between men and women [25, 26]. The available literature concerning any sex-differences for persistent HAIH has been inconsistent [6, 27–29]. We did not find any significant difference between males and females in the effect of a head injury on change in headache status from HUNT2 to HUNT3. In the analyses stratified upon sex, the positive associations from the combined analysis were observed to be significant only among men. This could be an incidental finding.

Earlier studies have shown an inverse dose-response relationship between the severity of head injuries and development of persistent headache [13, 14]. In our analyses the estimated odds for all unfavourable headache trajectories after a mild head injury were higher than the corresponding odds found for moderate head injury. This indicates that although sequelae in general after mild head injuries are milder compared to more severe head injuries, patients with previous mild head injuries may be just as, or even more affected by headache. This seems to confirm the paradoxical finding in earlier studies of a lack of a positive dose-response relationship between head injury severity and HAIH. However, the present study included few moderate head injuries, which gives low power. Furthermore, the classification of head injury severity has differed widely, which makes comparisons between studies difficult [30–32].

### Strengths

The major strengths of this study were the large population-based dataset on headache at two time points, combined with extensive objective information on each head injury, retrieved from medical records which eliminated recall bias regarding the head injury and enabled us to classify the head injuries according to severity. We had validated information about the headache status of the participants before the head injury, which enabled us to compare prevalence as well as frequency before and after the time of the head injuries and make comparisons with a non-exposed control group. This has not been possible in earlier studies. The design eliminates recall bias also regarding pre-study headache suffering, which can be a problem in prospective studies, as participants might tend to trivialise headache before the head injury because they understand their headache as a consequence of their head injury. Such possible under-reporting of pre-injury headache could be the reason why several prospective studies report pre-injury headache prevalence far below known headache prevalence in the general population [33–35]. This is especially a problem in studies without control groups. In some studies, using a control group with injuries other than head injuries could be an advantage, as one could theorize that physical trauma could cause headache through psychosocial stressors, regardless of which part of the body is injured. However, if the study is epidemiological, the design is population-based and the intention is to investigate whether head injury is a risk factor for development of headache regardless of the underlying mechanism, a community control group is more appropriate [36].

In a previous study, we analysed headache in HUNT3 related to all hospitalized head injuries (*n* = 940) between 1988 and HUNT3 [15]. As in the present study, there was a significant association between head injury and headache. However, in that study we were not able to take headache status before the trauma into account. This was a limitation because in patients with headache complaints a trauma may cause stress which can precipitate an attack of their pre-existing headache. Headache in a trauma setting can be a sign of a serious head injury, and these patients may therefore be referred to a hospital. Hence, one could imagine that pre-existing headache could act as a confounder. However, the present study shows that in the head injury group pre-existing headache was not more prevalent than in the control group, while new headache and exacerbation of headache was. We therefore find no reason to suspect the occurrence of such confounding.

### Limitations

The study was not designed to determine time of headache onset. Therefore, we cannot specify if the onset of headache was within 7 days after head injury, which is a criterion for classifying a headache as HAIH, according to ICHD-3 beta [10]. However, ICHD-3 beta states that this criterion is somewhat arbitrary and concludes further research is needed into which interval might be more appropriate [10].

The present study included only 32 individuals with a moderate and 35 individuals with a minimal head injury, which gives low power and uncertain results from these groups. Furthermore, generalization of results should be performed with caution, since only 56% of those invited to participate in HUNT2 and 42% of those invited to participate in HUNT3 answered the headache questionnaire.

Patients not examined at a hospital were not included. However, the proportion of patients with head injury being admitted to a hospital after examination by health care providers was larger in the period of data collection than it is today [26, 37]. This can mostly be attributed to increased availability of CT imaging and the implementation of guidelines for initial management of head injury in Norwegian hospitals [38].

### Implications for public health

HAIH is one of the most prevalent of secondary headaches worldwide and a potentially preventable one. Everywhere, but especially in low and middle-income countries, head injury is common and most often caused by road traffic injuries, falls and violence [39]. An important step in reducing the incidence of HAIH is therefore head injury preventive strategies [39].

The fact that development of HAIH also, or even especially, occurs after mild head injuries should have implications for the follow-up of patients with mild head injury. In Norway, like many other countries, there are no guidelines for follow-up after mild head injury. We know little about individual factors that predispose to development of persistent HAIH. We therefore suggest that all persons seeking medical advice due to a mild head injury should be encouraged to seek their general practitioner in the case of development of new headache or exacerbation of already existing headache with a duration longer than 3 months. A recent study suggests that a standardized tool might be helpful in the general population of concussion patients to assess for post-traumatic headache [40]. While we await better knowledge of how to best treat HAIH, we suggest using treatment strategies with proven efficacy against the primary headache that it most resembles [41].

### Future research

The incidence of mild head injury is high and a close follow-up of all persons experiencing a mild head injury would require a large effort for the health care service. It is therefore especially important to be able to identify persons at risk of developing HAIH and develop clinical guidelines for follow-up after mild head injury. HAIH occurred more frequently in patients with minimal traumatic intracranial haemorrhage after mild TBI than those without in a recent published study [42]. Another study found that persistent HAIH and migraine are associated with differences in brain structure [43]. Both studies suggests that it is possible to find underlying pathophysiology that separates HAIH from the primary headache type it phenotypically resembles. Future research should be aimed at understanding its pathophysiological mechanisms, acquiring knowledge on predictors for development of HAIH and based on this, develop effective preventive measures and treatment options.

## Conclusion

Individuals hospitalized due to a mild head injury were more likely to develop new headache suffering or report exacerbation of previously documented headache compared to the surrounding general population. Hence, the present study substantiates HAIH as a true secondary headache entity and not a primary headache misattributed to head injury.

## Abbreviations

BMI: Body Mass Index
CAGE: Modified Norwegian version of the CAGE alcohol-screening instrument
CI: Confidence Interval
HADS: Hospital Anxiety and Depression Scale
HAIH: Headache Attributed to Head Injury
HISS: Head Injury Severity Scale
HUNT: the Nord-Trøndelag Health Study
ICD: International Classification of Diseases
ICHD-3 beta: the International Classification of Headache Disorders, beta version of third edition
OR: Odds Ratio
